# *In vitro* co-culture models for the assessment of orthopedic antibacterial biomaterials

**DOI:** 10.3389/fbioe.2024.1332771

**Published:** 2024-02-05

**Authors:** Benedictus I. M. Eijkel, Iulian Apachitei, Lidy E. Fratila-Apachitei, Amir A. Zadpoor

**Affiliations:** Department of Biomechanical Engineering, Faculty of Mechanical Engineering, Delft University of Technology (TU Delft), Delft, Netherlands

**Keywords:** implant-associated infections, antibacterial biomaterials, *in vitro* co-culture models, osteogenic cells, immune cells

## Abstract

The antibacterial biofunctionality of bone implants is essential for the prevention and treatment of implant-associated infections (IAI). *In vitro* co-culture models are utilized to assess this and study bacteria-host cell interactions at the implant interface, aiding our understanding of biomaterial and the immune response against IAI without impeding the peri-implant bone tissue regeneration. This paper reviews existing co-culture models together with their characteristics, results, and clinical relevance. A total of 36 studies were found involving *in vitro* co-culture models between bacteria and osteogenic or immune cells at the interface with orthopedic antibacterial biomaterials. Most studies (∼67%) involved co-culture models of osteogenic cells and bacteria (*osteo-bac*), while 33% were co-culture models of immune cells and bacterial cells (*im-bac*). All models involve direct co-culture of two different cell types. The cell seeding sequence (simultaneous, bacteria-first, and cell-first) was used to mimic clinically relevant conditions and showed the greatest effect on the outcome for both types of co-culture models. The *im-bac* models are considered more relevant for early peri-implant infections, whereas the *osteo-bac* models suit late infections. The limitations of the current models and future directions to develop more relevant co-culture models to address specific research questions are also discussed.

## 1 Introduction

The effectiveness of antibiotics is diminishing due to the global proliferation of drug resistance, resulting in infections that are increasingly challenging to treat. This phenomenon, known as antimicrobial resistance (AMR), is responsible for the death of approximately 1.5 million individuals annually (World Health Organization, 2012; EARS-net, 2014; O’Neill, 2014; CDC, 2017; Burnham et al., 2019). If this trend continues in the upcoming decades, it will have a strong detrimental effect on the quality of life worldwide, particularly impacting medical procedures like surgery and implant replacements, as they will pose an increased risk of infection. Therefore, continued research on understanding, prevention, and treatment of AMR-related infections is crucial.

In the field of orthopedics, implant-associated infections (IAIs) can occur as bacteria (including multidrug-resistant ones) attach to and form a biofilm on the surface of an implant. This can lead to osteomyelitis, debilitating pain, severe inflammation, swelling, septic implant loosening, and, in the worst-case scenario, limb amputation or even death (Elek, 1956; Zimmerli et al., 1982; Masters et al., 2019; Saeed et al., 2019). These infections are difficult to cure, and for the currently used cementless orthopedic implants, there are no solutions to effectively prevent them. Consequently, it is essential to devise strategies to prevent biofilm formation on the implant surface. Many different surfaces are investigated for preventing bacterial adhesion, growth, and biofilm formation, which can primarily be categorized into bacteria-repellent, bactericidal and bacteriostatic surfaces (Modaresifar et al., 2019). The former type of surfaces prevents bacteria adhesion and, thus, inhibits biofilm formation. However, these surfaces may also repel host cells, which is not favorable for permanent bone implants, where implant osseointegration is vital for long-term implant survival, especially for cementless bone implants (Wang et al., 2011). In contrast, bacteriostatic and bactericidal surfaces can respectively suppress bacterial growth or kill bacteria by releasing antimicrobials, such as antibiotics (Cicuéndez et al., 2018), antimicrobial peptides (AMPs) (Boix-Lemonche et al., 2020a), or metal ions (Guo et al., 2017; Cochis et al., 2020; Shuai et al., 2024) into the immediate microenvironment for a certain period, or through contact killing effects induced by specific surface topographies (Izquierdo-Barba et al., 2015; Damiati et al., 2022).

For orthopedic applications, much of current research is directed toward finding biomaterials that can be both antibacterial and osteogenic. Over the past 2 decades, however, the importance of the interaction between the bone and immune cells, commonly referred to as osteoimmunology, has gained increased recognition and has been considered integral to the process of osseointegration (Takayanagi, 2007; Mountziaris et al., 2011; Loi et al., 2016; Mestres et al., 2021). Acute inflammation is known to be a critical initial step in bone healing (Marsell and Einhorn, 2011), and the communication that takes place between the immune and skeletal cells upon implantation plays a vital role in determining the eventual success or failure of the implant. Despite its significance, this interaction remains a largely overlooked and elusive aspect of research in the case of antibacterial biomaterials. The immune response to these biomaterials and its modulation may help in the prevention and treatment of IAIs (Dong et al., 2022). A successful novel biomaterial for orthopedic applications should be able to modulate the immune response toward bone regeneration while minimizing the chance of peri-implant infections (Feng et al., 2023). Toward this aim, suitable *in vitro* co-culture models are needed to study the potential of orthopedic biomaterials to orchestrate the interactions between the immune, stromal/osteoprogenitor, and bacterial cells.

This article provides a general overview of the currently available *in vitro* co-culture models used to assess the antibacterial properties of orthopedic biomaterials. The review is focused on the co-culture models involving bacteria with immune and/or osteogenic cells, and describes their main characteristics, methodology, results, and clinical relevance. The findings are discussed in the context of clinical requirements and the limitations of the current models. Finally, we present a perspective on further developments in this important area of research.

## 2 Results

### 2.1 Literature search output

**FIGURE 1:**
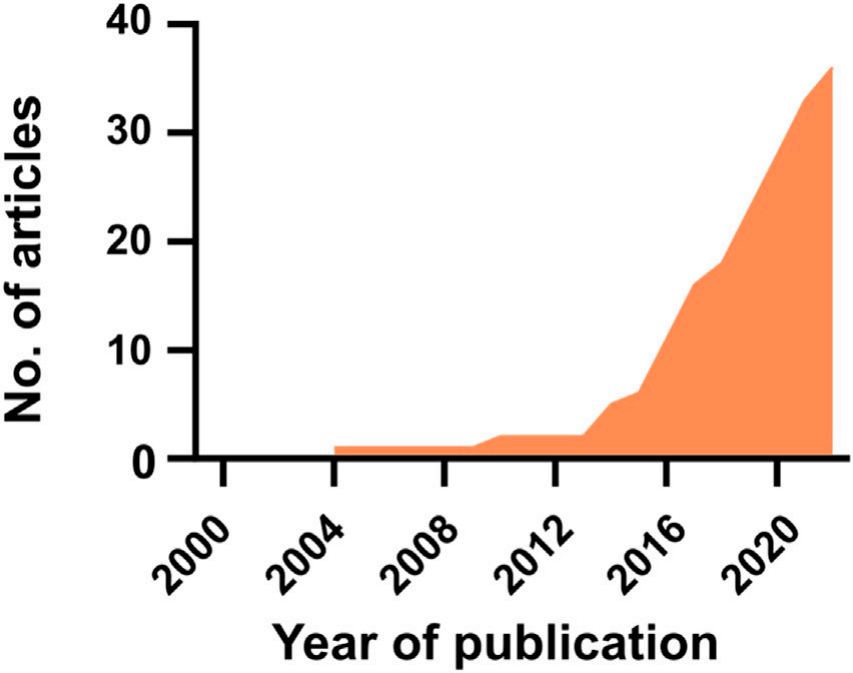
The articles published in this field from 2000 to 2023.

Based on the steps described in the Supplementary Material (Methods section), a total of 36 articles were found to fulfill the inclusion criteria (*i.e.,* research focused on *in vitro* co/tri-culture of bacteria with immune and/or osteogenic cells on orthopedic biomaterials). The first study was published in 2004, and since then, the interest has increased (Figure 1). The results of the literature search are detailed in Supplementary Tables S2–S4 (see Supplementary Material) and are summarized in the following sections.

### 2.2 Type of co-culture models and their characteristics

The different co-culture models found in the literature have been firstly divided based on the combination of the cells used in the model. After that, several culture conditions were further considered to characterize the different co-culture models. Namely, the type of bacterial, osteogenic, and immune cells investigated, the direct/indirect culture conditions, the cell seeding sequence, the ratio between the cells used in the co-cultures, the co-culture time, and the static or dynamic nature of the culture conditions.

**FIGURE 2:**
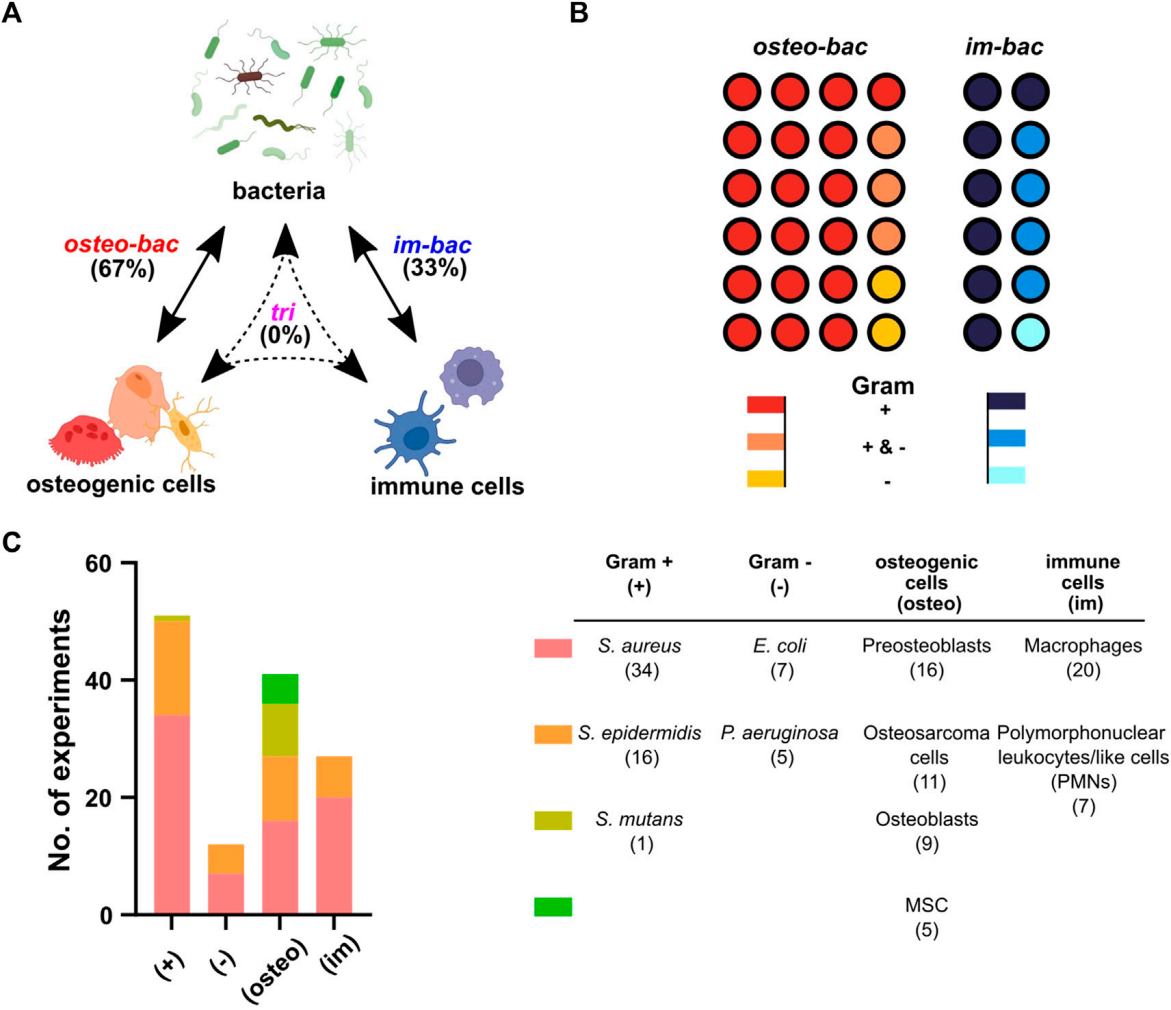
Co-culture models based on the involved cell type. **(A)** The terminology used in this review for the different co-culture models: *in vitro* models concerning osteogenic and bacteria (osteo-bac), immune cells and bacteria (im-bac) or all three in a tri-culture (tri). Created with BioRender.com **(B)** The distribution of the different co-culture models between the 36 articles. A distinction has been also made between the studies using Gram-positive (+) bacteria, Gram-negative (−) bacteria, or both (+&−). **(C)** An overview of cell types used in the experiments together with the specific cells per type.

All the included studies used the co-culture of bacteria with either osteogenic or immune cells. No tri-culture model was found (Figure 2A). Therefore, co-culture models were categorized into two different groups: (1) co-culture of osteogenic and bacterial cells (*osteo-bac*) and (2) co-culture of immune and bacterial cells *(im-bac*). The *osteo-bac* models were the most frequently encountered (∼67%).

As expected, the majority of the articles focused on Gram-positive bacteria (Figure 2B). The most commonly researched Gram-positive bacteria were *Staphylococcus aureus* (34 experiments), followed by *Staphylococcus epidermidis* (16 experiments), and *Streptococcus mutans* (1 experiment) (Figure 2C). Three articles focused only on the Gram-negative and seven on both Gram-positive and Gram-negative bacteria. The Gram-negative bacteria examined included *Escherichia coli* (7 experiments) and *Pseudomonas aeruginosa* (5 experiments). Regarding the osteogenic cells, preosteoblasts were the most frequently encountered cells. As for the immune cells, macrophages were the most prevalent cell types (Figure 2C).

Notably, all the studies were based on direct co-culture models. Interestingly, in the *osteo-bac* co-culture models, half of the studies used a host cell medium (αMEM or DMEM), whereas the other half used a combination of a host cell medium (αMEM or DMEM) and a bacterial-cell medium (Mueller Hinton Broth, Luria Bertoni, NB Basal Medium or Todd Hewitt Broth), with the proportion of the bacterial-cell medium ranging between 2% and 50%. In comparison, all of the *im-bac* studies used a host cell medium (RPMI-1640, IMDM, αMEM or DMEM), except for one study which used heparinized blood to assess its effects on the viability, phagocytic activity, and tissue regeneration capabilities of polymorphonuclear neutrophils (PMNs) as compared to the standard culture medium (RPMI-1640) (Guo et al., 2017).

**FIGURE 3:**
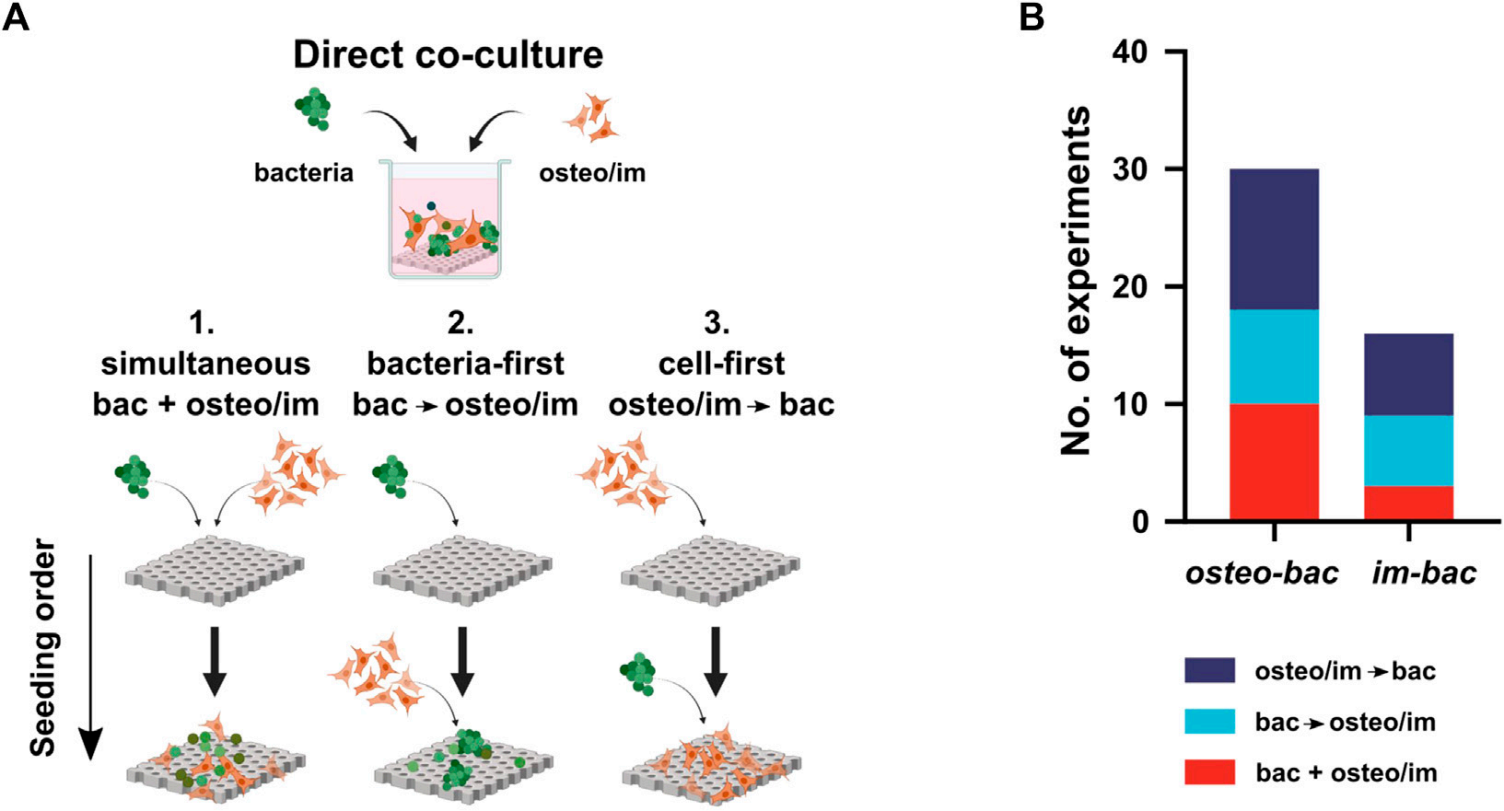
**(A)** The direct co-culture models use three different seeding sequences of bacteria and host cells: 1. Simultaneous (bac + osteo/im), 2. Bacteria-first, (bac→osteo/im), and 3. Cell-first (osteo/im→bac). Created with BioRender.com. **(B)** The distribution of the seeding sequences among the experiments.

Further characterization of these models was based on the cell seeding sequence and the ratio between both types of cells used in the cultures. Regarding the seeding order (Figures 3A, B), three different combinations were encountered: simultaneous seeding of both cell types (bac + osteo/im), bacteria-first (bac→osteo/im), and osteo/immune cell-first (osteo/im→bac). Most of the studies used a cell-first seeding sequence (19 studies). Alternative terms were associated with these different seeding methods to reflect some clinical scenarios. Namely, “competition” for the simultaneous seeding, “prevention” for bac→osteo/im sequence, and “protection” (of adhered host cells) for the osteo/im→bac sequence. Alternatively, the terms “race for the surface,” “perioperative,” and “post-operative” infection models were used to describe the same concepts, respectively (Yue et al., 2014; Deshmukh et al., 2016; Cochis et al., 2020).

**FIGURE 4:**
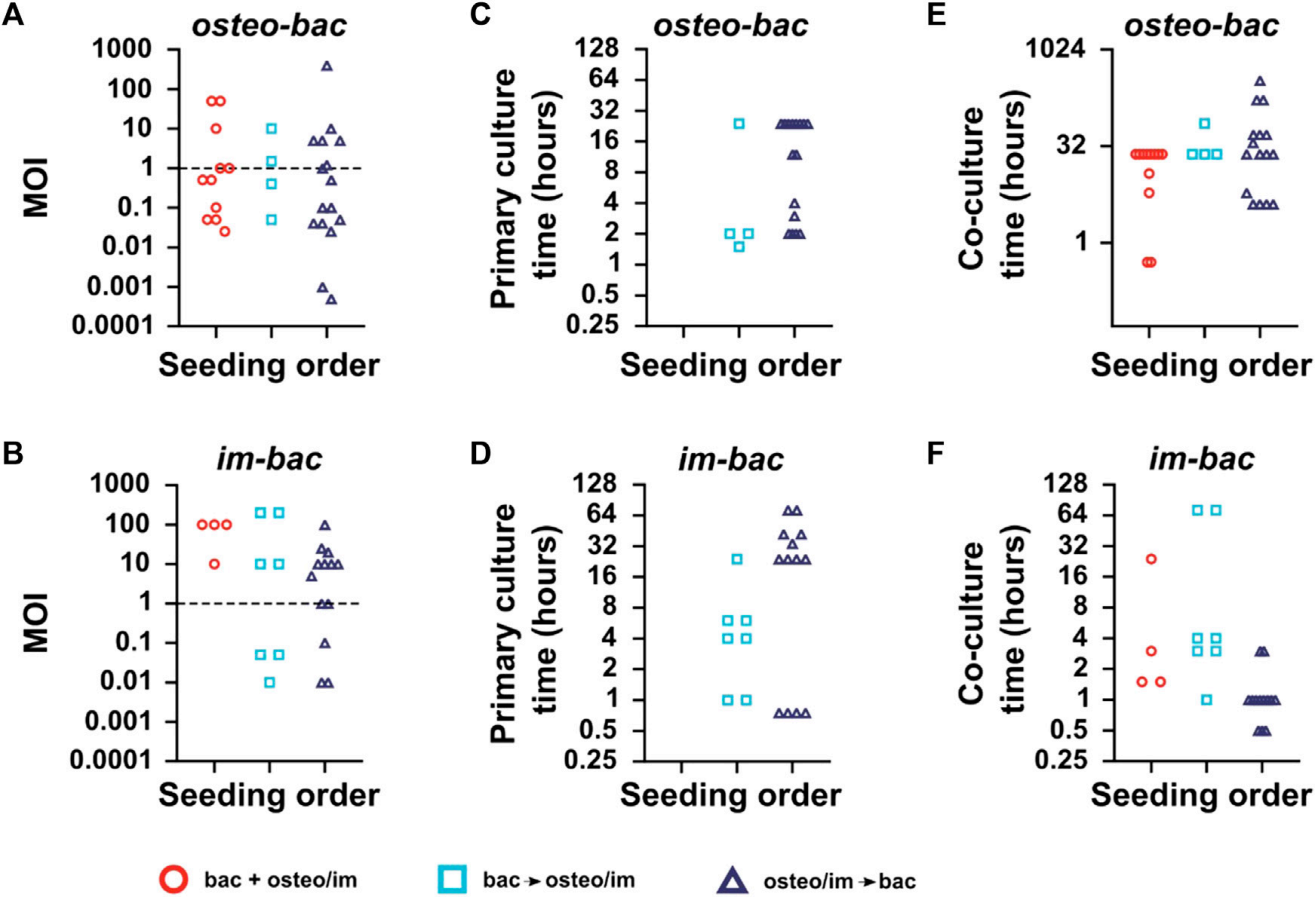
**(A)** The multiplicities of infection (MOIs), the ratio of bacteria to host-cells used in the direct co-culture models for each seeding sequence for *osteo-bac* and **(B)** *im-bac* models. **(C)** The primary culture time (i.e., the initial culture time for the first cell type seeded on the biomaterial). for *osteo-bac* models and for **(D)** *im-bac* models. **(E)** The co-culture time (i.e., the total co-culture time for both cell types on the biomaterial) for *osteo-bac* models and for **(F)** *im-bac* models. Studies involving the usage of dynamic co-culture models or experiments in which the MOI, primary culture time, or co-culture time was not mentioned are excluded.

The multiplicity of infection (MOI), particle-to-cell ratio, or infection ratio were used to define the ratio of the bacteria to the host cells in the co-culture (Wagner and Bryers, 2004; Svensson et al., 2014; Ghimire et al., 2016; Li et al., 2017; Mendoza et al., 2017; Zaatreh et al., 2017; Chu et al., 2018; Ellett et al., 2019). The MOI was calculated for all the included studies to shed light on the MOI trends between the models (Figure 4A). Remarkably, the MOIs values in the *osteo-bac* models were commonly within the range of 0.025–50 (∼90%), with very few exceptions (Ghimire et al., 2016; Mohiti-Asli et al., 2016; Cicuéndez et al., 2018; Sánchez-Salcedo et al., 2023). The MOIs observed in *im-bac* models were between 0.01 and 200 with most of the studies using a MOI between 1 and 100 (Figure 4B). In the *im-bac* models using simultaneous seeding, MOIs were always in the range of 10–100.

Regarding the culture time, a distinction was made between the primary culture time and co-culture time. The former refers to the culture time of the first seeded cell type while the latter refers to the co-culture time. In the case of *osteo-bac* models (Figure 4C), the primary culture times varied between 0.5 and 24 h. The primary culture time of *im-bac* models (Figure 4D) was between 0.75 and 72 h.

The co-culture durations for the *osteo-bac* models varied depending on the culture sequence and were in the ranges of 0.5–24 h, 24–72 h, and 4 h up to 14 days for simultaneous, bacteria-first, and cell-first seeding, respectively (Figure 4E). These differences in the co-culture time were influenced by the specific research questions investigated in the studies. Some studies focused on the initial adhesion of bacteria, which could be examined within 30 min (Foss et al., 2015), while others assessed the long-term osteogenic modulation of highly antibacterial surfaces, requiring a co-culture duration of 14 days (Mohiti-Asli et al., 2016). In the case of the *im-bac* models (Figure 4F), the co-culture time did not exceed 72 h. For simultaneous seeding, the time varied between 1.5 and 24 h, while for bacteria-first seeding, the range extended from 1 to 72 h. In the case of the cell-first models, the primary culture duration spanned from 0.5 to 4 h. The shorter co-culture times in the cell-first studies can be explained by the focus of those studies on the immediate immunomodulatory effect of the biomaterial upon seeding. However, in the study by Zwicker and co-workers (Zwicker et al., 2022), the co-culture time was extended to 72 h to observe a polarization shift in the macrophages. No such shift was, however, observed in the co-culture while it did occur in their monoculture experiments.

Most co-cultures (∼85%) were conducted under static conditions. Among the four studies utilizing dynamic co-culture conditions, two involved *osteo-bac* co-culture models while the other two were *im-bac* models, all four of which used a bacteria-first sequence (Lee et al., 2011; Gu et al., 2014; Yue et al., 2014; Ellett et al., 2019). Interestingly, the two *osteo-bac* models focused on the effect of shear stress in lab-on-a-chip (LOC) devices, whereas only one *im-bac* model focused on the effect of flow on macrophage mobility and the other on neutrophil migration under static conditions in a LOC, to minimize the effect of fluid shear stress on cell behavior.

### 2.3 Effects of osteogenic and immune cells on the antibacterial properties of orthopedic biomaterials

#### 2.3.1 Osteo-bac models

The primary research focus of the studies using the *osteo-bac* co-culture models has usually been to investigate the antibacterial effectiveness of the biomaterials in the presence of bone-like cells (Supplementary Table S2). This has been mainly assessed by looking at the surface coverage of both cell types.

Cell-first studies (*i.e.*, when preosteoblasts were seeded first) (Zaatreh et al., 2016; Zaatreh et al., 2017; Tran and Tran, 2021) revealed interesting insights regarding the interaction of both cell types. Whereas the silver (Ag) nanoparticles present on the surface of titanium specimens prevented biofilm formation during bacterial monoculture, during co-culture using the cell-first sequence, *S. aureus* survived on top and inside preosteoblasts (MC3T3-E1), using them as a shield against the antimicrobial surface (Tran and Tran, 2021). This behavior decreased both the viability of osteogenic cells and the antibacterial efficacy of the biomaterial. The same trend has been observed for *S. epidermidis* in contact with a titanium surface with a rapidly corroding magnesium layer. While the coated-titanium specimens exhibited antibacterial behavior during bacterial monoculture, they were ineffective during cell-first *osteo-bac* co-culture (Zaatreh et al., 2016; Zaatreh et al., 2017).

When *S. aureus* was seeded first, higher preosteoblast (MC3T3-E1) death, as compared to simultaneous and cell-first experiments, was observed for a biomaterial consisting of hydroxyapatite (HA) to promote preosteoblast adhesion and chitosan to inhibit bacterial survival (Chu et al., 2018). Even with a low MOI (1:20), the antibacterial properties of this biomaterial were not strong enough to prevent bacteria from adhering to its surface and starting to form biofilms. This resulted in preosteoblasts not being able to attach to the biomaterial surface and proliferate.

In contrast to the static conditions, the dynamic perfusion systems allowed for a method for assessing the live behavior of bone cells during healing with and without the presence of a microbial threat. To illustrate the progression of wound healing, the effects of flow on the bone formation of MC3T3-E1 preosteoblasts were assessed. In addition, the administration of *S. epidermidis* into the channel led to the development of biofilm and adversely affected the osteogenic differentiation of the cells inside this 3D flow system (Lee et al., 2011). Another study, using osteosarcoma cells, also utilized a flow system to assess the surface coverage by osteosarcoma cells and *S. epidermidis* (MOI = 1:1) on (non-) anodized Ti and Ti6Al4V surfaces (Yue et al., 2014). Generally, the dynamic culture conditions facilitated a better distribution of bacteria at the beginning of each experiment, negatively impacting the survival of human osteosarcoma cells on non-anodized surfaces. The anodized surfaces proved to be successful in inhibiting bacterial growth and improving host cell attachment and surface coverage.

In all of the aforementioned studies, different methods were utilised for the assessment of bacterial and host cell survival on top of the implants. A basic method used for assessing the antibacterial efficiency of a biomaterial in the presence of osteogenic cells was by counting the colony-forming unit (CFU) after removing adhered bacteria from the biomaterial by sonication (Supplementary Table S3) (Lee et al., 2011; Mohiti-Asli et al., 2016; Jia et al., 2021). To compare the survival of both cell types, several studies detached the mammalian cells from the surface with trypsin, followed by cell counting using Trypan blue and a cell counter (Foss et al., 2015; Ghimire et al., 2016; Zaatreh et al., 2016; Zaatreh et al., 2017; Martínez-Pérez et al., 2019; Cochis et al., 2020). The surface coverage or morphological changes in both cell types was determined by scanning electron microscopy (SEM) (Foss et al., 2015; Jia et al., 2016; Mohiti-Asli et al., 2016; Zaatreh et al., 2016; Zaatreh et al., 2017; Chu et al., 2018; Qiu et al., 2019; Shen et al., 2020; Tan et al., 2020; Rivera et al., 2021; Damiati et al., 2022), optical microscopy (Lee et al., 2011; Jia et al., 2016; Tran and Tran, 2021), and fluorescence microscopy (Yue et al., 2014; Foss et al., 2015; Jia et al., 2016; Chu et al., 2018; Cicuéndez et al., 2018; Martínez-Pérez et al., 2019; Qiu et al., 2019; Tao et al., 2019; Boix-Lemonche et al., 2020b; Boix-Lemonche et al., 2021; Jia et al., 2021; Sánchez-Salcedo et al., 2023).

In addition to assessing host or bacterial cell survival in terms of cell number or morphological features, several studies investigated the invasion rate of bacteria, to highlight the infection rate of bacteria on different substrates. Different methods were employed to quantify the number of internalized bacteria inside bone cells, as a form of determining the invasion rate of bacteria into the host cells. Fluorescence imaging has been employed using cytoskeletal and nucleus staining combined with pre-stained bacteria (Yue et al., 2014; Foss et al., 2015; Jia et al., 2016; Chu et al., 2018; Cicuéndez et al., 2018; Qiu et al., 2019; Tao et al., 2019; Boix-Lemonche et al., 2020b; Boix-Lemonche et al., 2021; Jia et al., 2021). Additionally, acridine orange has been utilized to stain bacterial DNA and visualize the internalization of bacteria by the osteogenic cells (Chu et al., 2018; Martínez-Pérez et al., 2019; Sánchez-Salcedo et al., 2023). Another technique to quantify internalized bacteria involves destroying the cellular membrane to release all the internalized bacteria, known as the “Gentamicin protocol” (Jia et al., 2016; Tran and Tran, 2021). This method entails removing all extracellular bacteria using gentamicin or PBS, lysing the cells to release intracellular bacteria, and performing CFU counting or live/dead staining of the released bacteria.

Another technique for assessing cell death or bacterial invasion was by measuring lactate dehydrogenase (LDH), which is released by host cells when they die (Chu et al., 2018; Cicuéndez et al., 2018). Notably, one study employed qPCR to quantitatively assess biofilm formation after 24 h of co-culture (Chu et al., 2018). By sonication of the biomaterials in PBS, the biofilm was collected and biofilm-specific genes (*i.e.*, icaA, icaD, hld, and spa) were measured. Finally, the osteogenic response of host cells to bacteria and biomaterials has been evaluated to a limited extent using ALP, OCN (Shen et al., 2020), RUNX2 (Damiati et al., 2022), and ARS staining (Lee et al., 2011; Mohiti-Asli et al., 2016).

#### 2.3.2 Im-bac models

All the *im-bac* co-culture studies, except for one, focused on the recruitment and pro-inflammatory response of immune cells (PMNs and macrophages) and how the biomaterial affects this response in a co-culture model (Supplementary Table S2).

Yang *et al.* (Yang et al., 2021) showed the effects of the three different seeding sequences, all using the same MOI (10:1), with *S. aureus* and Raw 264.7 macrophages on UV/ozone irradiated antibacterial titanium surfaces (Ti-UV). The strongest antibacterial effects, as determined by the increased reactive oxygen species (ROS) production of macrophages in combination with more internalized bacteria, were achieved when macrophages were seeded first on the biomaterial surface followed by bacterial seeding after 24 h. In the case of the simultaneous and bacteria-first model, the macrophages did not have adequate space to survive and proliferate on the surface, leading to a reduced rate of bacterial killing by macrophages.

One interesting aspect of this study was that during the monoculture of macrophages on the Ti-UV surface, expression of pro-inflammatory genes (TNF-α and IL-6) were downregulated, whereas IL-10 and Arg-1 were upregulated, indicating a reduced foreign body response (FBR). Reduced early production of excessive ROS by macrophages in response to the Ti-UV surface helped create a favorable environment for the macrophages as compared to Ti. This controlled regulation of ROS production facilitated and enhanced the antibacterial and phagocytic activity of macrophages against bacteria.

By modulating the macrophage response in this way, the Ti-UV surface demonstrated potential benefits for promoting a favorable immune response, reducing the foreign body response, and potentially improving the biocompatibility of this biomaterial for this application.

In addition, this was the only study which also tested the osteoimmunomodulatory effect of the Ti-UV biomaterial on MSCs. They found that the conditioned medium (CM) from LPS-stimulated macrophages cultured on this biomaterial improves the osteogenic response of the MSCs.

Only one *im-bac* study has directly focused on macrophage polarization to limit the foreign body response toward a contact-killing surface instead of focusing on enhanced phagocytosis (Zwicker et al., 2022). The monoculture of THP-1 stimulated macrophages on Ti6Al4V, with and without Poly (hexamethylene) biguanide hydrochloride (PHMB) coating, showed that the layer did not induce a pro-inflammatory response. During bacteria-first co-culture, this material enabled macrophage attachment, viability, and improved tissue regeneration through early killing of the bacteria.

In two *im-bac* studies, LOC devices were developed with a focus on achieving high-output systems capable of simultaneous handling of a large number of experiments (Gu et al., 2014; Ellett et al., 2019). These devices were specifically designed to enhance the throughput and efficiency of the experiments. For example, a chip was created with a chitosan and glass-patterned surface to reveal macrophage mobility and phagocytic activity in response to bacterial presence (Gu et al., 2014). Under a constant flow to refresh the culture media, it was seen that the macrophage mobility was increased on chitosan as compared to glass, which reduced the bacterial presence on chitosan. Using a patterned surface, multiple parts on the same chip were used to compare the differences between macrophages on both surfaces. Finally, another LOC device allowed for quick and easy assessment of neutrophil recruitment to bacteria (Ellett et al., 2019). This device consisted of multiple smaller inner and one large outer compartment, which were only connected with a channel. The recruitment of neutrophils from the outer part toward the inner compartments, in which bacteria were present, was assessed using ROS staining.

Similar to *osteo-bac* models, the combination of CFU count (Deshmukh et al., 2016; Chen et al., 2017) with cell count (Wagner and Bryers, 2004; Gu et al., 2014; Svensson et al., 2014; Guo et al., 2017; Reigada et al., 2020; Zwicker et al., 2022) has been the most commonly used approach to evaluate the antibacterial effectiveness of biomaterials in the presence of the immune cells (Supplementary Table S4).

SEM imaging to see lysed cells or bacteria on the surface or on top of immune cells was a commonly used method to assess the survival of immune and bacterial cells (Svensson et al., 2014; Mendoza et al., 2017; Hou et al., 2019; Reigada et al., 2020; Yang et al., 2021). The staining of both macrophages and bacteria, followed by confocal microscopy (Zwicker et al., 2022) or, in some cases, pre-staining of bacteria (Wagner and Bryers, 2004; Chen et al., 2017) or flow cytometry (Chen et al., 2017; Mendoza et al., 2017), have been used to assess the number of internalized bacteria inside macrophages. In addition, the previously mentioned gentamycin protocol has also been used to determine the rate of phagocytosis by macrophages (Li et al., 2017; Mendoza et al., 2017), instead of determining the rate of invasion, as was the case in the *osteo-bac* studies.

Remarkably, while LDH measurements were used to determine the number of lysed host cells in *osteo-bac* models, it was used to determine bacterial death in *im-bac* models (Svensson et al., 2014; Guo et al., 2017). A final method of assessing phagocytosis was by looking at the ROS activity of the immune cells while being co-cultured with bacteria. The level of oxidative stress inside the immune cells is a direct indication of an enhanced pro-inflammatory response (Wagner and Bryers, 2004; Svensson et al., 2014; Ellett et al., 2019; Yang et al., 2021).

Only a few studies have looked into the effects of biomaterials on immune response by measuring the expression of pro- (IL-1β, TNF-α, IL-6) and anti- (IL-8, IL-10) inflammatory markers, using qPCR or ELISA (Wagner and Bryers, 2004; Svensson et al., 2014). One study has specifically tried to use these markers to assess the polarization shift after 1, 2, and 3 days of co-culture (Zwicker et al., 2022).

## 3 Discussion

Assessment of antibacterial biomaterials under more relevant clinical conditions requires novel *in vitro* culture models that incorporate different cell types, ECM components, and soluble factors. Here, we reviewed the co-culture models currently used for the investigation of orthopedic antibacterial biomaterials. It is worth noting that the early results published at the beginning of the 2000s used *im-bac* models. Later, the *osteo-bac* models started to be also used and quickly exceeded the *im-bac* models in number. The slow pace of new studies in this field indicates a lack of recognition within the biomedical community regarding the magnitude of this issue. It underscores not only the necessity for developing better biomaterials but also emphasizes the requirement for enhanced *in vitro* investigations to narrow the gap between biomaterial development and clinical applications. The knowledge resulting from such models has created new frontiers in the study of cell-surface and cell-cell interactions, which could impact the future design of orthopedic antibacterial biomaterials. In particular, the dominant paradigm is expected to shift from dual (*i.e.,* osteogenic and antibacterial) functionalities to a triple-biofunctionality paradigm encompassing immuno-modulatory, osteogenic, and antibacterial responses. Such a more comprehensive paradigm is likely to improve the prevention and treatment of implant-associated infections particularly in the light of the emergence of antibiotic-resistant strains. Furthermore, the findings of the included studies point toward new approaches and methodologies that may improve the current models and their output, by more strongly embracing emerging technologies, such as microfluidics, 3D printing, and the use of advanced bioreactors.

The results of this review revealed that all the co-culture models used to date are based on direct cultures of bacteria with either osteogenic or immune cells. In general, the direct co-culture models are considered more relevant than the indirect co-culture models, primarily because the cells are subjected to both physical and paracrine interactions during the cell culture experiments (Hayrapetyan et al., 2016; Sakthivel et al., 2019). This is also true for co-culture models involving bacteria and host cells, where direct interactions between these cell types are critical in defining the effectiveness of the biomaterial concerning the viability and functional efficacy of host cells as well as the targeted antibacterial biofunctionality. It is, therefore, not surprising that no studies have used indirect co-culture models. Nevertheless, indirect co-culture models may be of relevance when addressing specific research questions, such as how to delineate the effects of an antibacterial agent released from the biomaterial surface from the contact-killing effects, as shown in a study by Sánchez-Salcedo *et al.* (Sánchez-Salcedo et al., 2023). The indirect osteo-immunomodulatory effects of an *im-bac* co-culture on MSCs is another such example (Yang et al., 2021).

**FIGURE 5:**
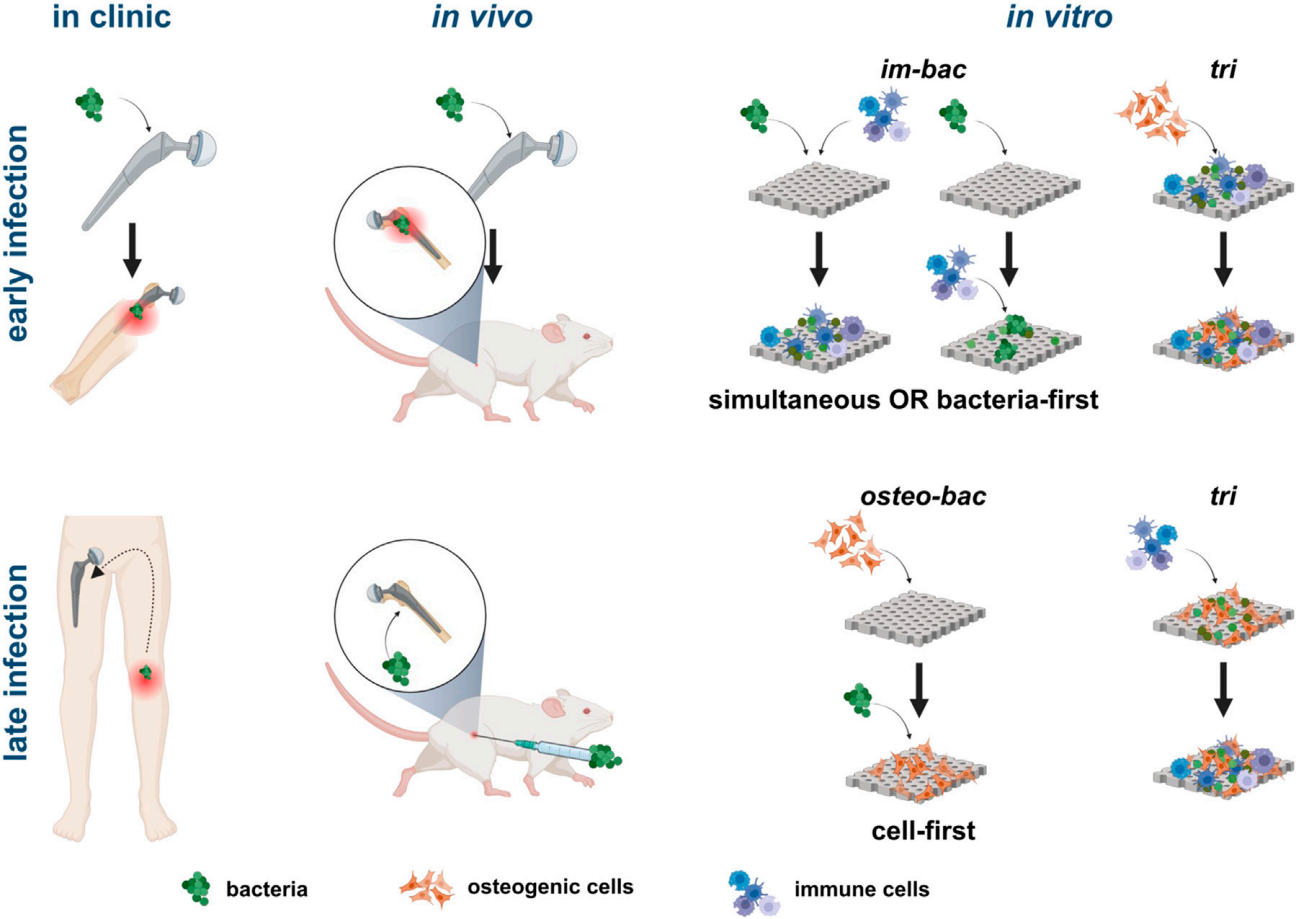
Illustration of clinical and *in vivo* scenarios directly related to different *in vitro* co-culture models. For simulating early infections, models where bacteria and immune cells are introduced simultaneously or with bacteria first are more relevant. The addition of bone-forming cells in a triple culture model can simulate the progression of the infection in the peri-implant area. For late-stage infections, seeding bone cells first before adding bacteria is more clinically relevant with the subsequent addition of immune cells in a triple culture adding complexity to the system. Created with BioRender.com.

The selection of the direct co-culture models should be based on the research questions addressed. From a biomaterial perspective, it may be more relevant to make a distinction between early and late IAIs (Trampuz et al., 2005; Ribeiro et al., 2012; Cochis et al., 2020) and develop (co-culture) models that address each specific type of infection (Figure 5). The micro-environment *in vivo* is far more complicated than *in vitro*. Focusing on a specific type of infection may enable researchers to better mimic the most relevant aspects of *in vivo* conditions in their *in vitro* co-culture models. The phases following implantation are hemostasis, inflammation, tissue repair, and tissue remodeling (Marsell and Einhorn, 2011; Ribeiro et al., 2012; Carnicer-Lombarte et al., 2021). An early *in vitro* infection model should, therefore, include bacteria and inflammatory cells (*im-bac* models) with the possible addition of blood components (to represent the early hemostasis phase), followed by the addition of stromal/progenitor cells at a later time point. Until now, only a few studies have focused on the hemostasis phase and the effects of serum or blood on the antibacterial efficiency of a biomaterial, even though the addition of blood provides a clinically relevant microenvironment, which is beneficial for reducing the discrepancy between *in vitro* and *in vivo* outcomes (Wagner and Bryers, 2004; Guo et al., 2017; Li et al., 2017; Hou et al., 2019).

The inflammatory response following the hemostasis phase during an early infection can be best mimicked by *im-bac* models in which the macrophages will be present within hours after infection (Martinez, 2008). These time points are also seen in the included *im-bac* models, where the focus has been on the pro-inflammatory response during this initial inflammatory response toward a foreign body or bacterial infection (Arciola et al., 2012). Longer co-culture (>7 days) for macrophages may result in automatic phenotype change toward M2, altering their behavior and function (Chamberlain et al., 2015). Overall, optimal culture times depend on the bacterial and immune cell types used, each strain having distinct proliferation times, and the research question addressed.

Although the seeding sequence can be established based on the type of infections addressed, the *in vitro* results reviewed here reveal a few interesting findings related to the effects of the immune cells on the bacteria during co-culture experiments. In *im-bac* cell-first models, for example, despite enough room being available for bacteria, fewer *S. aureus* adhered on the biomaterial surface as most were attached to or were found inside macrophages. While *im-bac* cell-first models may not have direct relevance to *in vivo* investigations, they hold significant potential in understanding the immunomodulatory effects of biomaterials and their implications for antibacterial efficacy. As for the MOI, a low MOI is more clinically relevant for early infections as compared to high MOIs that are more representative of late infections (Fitzgerald, 1979; Ribeiro et al., 2012). In addition, a low MOI also helps researchers to achieve successful bacteria-first co-culture, as the early adhesion and surface coverage of bacteria can create a cytotoxic microenvironment and compromise the adhesion of host cells to the biomaterial while also adversely affecting the proliferation of such cells (Yue et al., 2014).

In the case of late infections, the bone tissue is already present on the implant surface. The co-culture models should, therefore, include bone/osteoprogenitors cells, bacteria, and the (vascularized) bone matrix, followed by the addition of immune cells within a few hours after the onset of the infection. The *osteo-bac* models combined with a cell-first seeding sequence can closely mimic this situation. Furthermore, in post-operative infections, high MOIs can be expected since the bacterial load could go toward 10^4^–10^6^ bacteria (Fitzgerald, 1979; Ribeiro et al., 2012).

The primary culture time of the *osteo-bac* models reviewed here did not exceed 24 h, meaning that no bone formation could have taken place. Such experiments are, however, crucial in initial assessments of the effectiveness of antibacterial surfaces. While in some cases the biomaterial is observed to suppress the biofilm formation in monocultures, bacteria were observed on top of osteogenic cells in the co-culture assessments of the same biomaterials (Zaatreh et al., 2016; Zaatreh et al., 2017; Tran and Tran, 2021). This phenomenon could be due to the preference of bacteria for attaching to eukaryotic surfaces rather than to biomaterials (Alkow and Portnoy, 1992). Knowledge of this bacterial survival mechanism is essential because the bacteria attached to host cells may survive even if the substrate has antibacterial properties (Tran et al., 2020). The common perspective of assessing the “race for the surface” does not apply in such cases (Gristina et al., 1989). The battle is not only for the surface but also for survival, as adhered osteogenic cells alone may not effectively combat bacterial infections. This highlights the importance of having surfaces that can both directly kill the bacteria on their surface and release antibacterial agents in the bone tissue surrounding the implant without affecting the viability of the host cells. Such observations could have major implications for the future directions of biomaterial research. The addition of immune cells or using an early infection model followed by the addition of osteoprogenitor cells could better mimic the battle for survival.

In future co-culture models of both early and late infections, immune cells from the innate and/or adaptive immune system should be considered. Such an approach will enable us to better understand and harness the endogenous antibacterial and regenerative potential of the host organism. Such advanced models can lead to a reduction of animal studies while shedding light on relevant fundamental questions related to IAIs.

Based on the recent fundamental knowledge generated in the field, investigations using relatively less complex models may be helpful to investigate, for example, the role of immune cells as antibacterial “drugs,” the surface-induced pro-inflammatory effects for a controlled duration, and the phagocytosis *vs* invasion processes. While internalized bacteria inside immune cells can indicate successful phagocytosis (Yang et al., 2021), they could also suggest successful invasion (Li et al., 2017; Mendoza et al., 2017). CFU counting of internalized bacteria in lysed macrophages can be argued to assess the incapability of macrophages to eradicate the threat, as the bacteria are still alive inside them, thus measuring the invasion rate (Mendoza et al., 2017). Conversely, a reduction in the number of invading bacteria may be due to the biomaterial’s success in managing cell invasion (Li et al., 2017). Understanding such aspects could lead to novel design criteria for antibacterial biomaterials.

Additional possibilities to scavenge the bacteria from or kill them within the tissue are relevant and may include the modulation of the response of the immune cells to maximize their antibacterial function. The results of the included co-culture models seem to indicate that the surface/biomaterial antibacterial effects are surmounted by cell-cell communication. More detailed investigations are, however, needed to elucidate the effects of the antibacterial surface *per se* in such models. The existing co-culture models should, therefore, be further improved to accommodate relevant tissue components and microenvironmental conditions (such as flow) for each infection type. This could be achieved by using emerging technologies such as 3D bioprinting of microtissues, organ-on-chip models, advanced bioreactors, and perfusion systems. In addition, advanced single-cell studies and the associated methodologies offer new tools for fundamental understanding of cell-biomaterial interactions. Applying these tools to novel and relevant co- and tri-culture models is essential for the assessment of multifunctional biomaterials and should become part of the research methodology used for developing such biomaterials. This approach will not only reveal unprecedented fundamental knowledge on the working mechanism of such biomaterials but will also contribute to decreasing the dependence on *in vivo* models thereby fostering the research towards the clinics. Future orthopedic antibacterial biomaterials should be able to prevent both early and late IAIs. This may require more advanced concepts, such as on-demand drug delivery upon external/endogenous stimuli. Such advancements are to be expected in the near future.

## 4 Conclusion

The development of multifunctional biomaterials presents significant challenges in identifying suitable *in vitro* culture models to effectively evaluate their capacity to regulate immune responses, enhance bone regeneration, and prevent peri-implant infections. This review focused on the co-culture models currently used for the assessment of the efficacy of antibacterial orthopedic biomaterials. The findings have revealed the use of either *im-bac* (co-culture of immune cells and bacteria) or *osteo-bac* (co-culture of osteogenic cells and bacteria) models, all of which have been direct co-culture models. The seeding sequence employed in these models was different depending on the type of peri-implant infection investigated (*e.g.*, early *vs.* late infections). The *im-bac* models are considered better suited for the study of early peri-implant infections, while *osteo-bac* models are particularly relevant to late infections. Future developments of such co-culture models are expected to include the incorporation of more relevant components and cell types.
