# Supplementary material for: In vitro co-culture models for the assessment of orthopedic antibacterial biomaterials
Source: Front Bioeng Biotechnol. 2024 Feb 5;12:1332771. doi: 10.3389/fbioe.2024.1332771 (PMC10875071; doi:10.3389/fbioe.2024.1332771)
Supplement: Supplementary file 1 [file Table1.docx]

Supplementary document to

*In vitro* co-culture models for the assessment of orthopedic antibacterial biomaterials

Benedictus I.M. Eijkel^1*^, Iulian Apachitei^1^, Lidy E. Fratila-Apachitei^1*,^ Amir A. Zadpoor^1^

*^1^ Department of Biomechanical Engineering, Faculty of Mechanical Engineering,* *Delft University of Technology (TU Delft), 2628 CD, Delft, Netherlands*

*^*^Corresponding authors:*

*B.I.M. Eijkel, E-mail:* *B.I.M.Eijkel@tudelft.nl*

*L.E. Fratila-Apachitei, Email: E.L.Fratila-Apachitei@tudelft.nl*

**Supplementary methods**

The primary search databases used in this study were Web of Science and Scopus. Keywords and their relevant synonyms included: (Bacteria) AND (Bactericidal OR Antibacterial) AND (Osteo OR Immune OR Osteoimmunology) AND (Co-culture) AND (Orthopedic biomaterial) (Table S1). The initial search yielded 299 initial results, including duplicates. Therefore, inclusion and exclusion criteria were applied to filter the articles during the first appraisal stage. Firstly, publications that were duplicates were removed from the list. Subsequently, articles were screened based on their title and abstract, and review papers, conference papers, or book chapters were excluded. Next, articles meeting the following four criteria were included: 1) the study involved *in vitro* co/tri-culture models; 2) the study investigated the interaction between immune/osteogenic cells and bacterial cells (co-culture) and/or immune-osteogenic-bacterial cells (tri-culture); 3) the study involved an orthopedic biomaterial; 4) the study focused on bacterial cells that cause orthopedic implant-associated infections. This comprises *Staphylococci* (~ 65 %), *Pseudomonas* (~ 10 %) and, to a smaller extent, *Enterococci*, *Streptococci* and *Escherichia. coli* (Ribeiro et al., 2012). In the end, 36 articles fulfilled these criteria and therefore were selected and discussed in this review.

**Supplementary tables**Table S1: An overview of all the keywords, (Bacteria) AND (Bactericidal OR Antibacterial) AND (Osteo OR Immune OR Osteoimmunology) AND (Co-culture) AND (Orthopedic biomaterial) and their relevant synonyms used for searching the Web of Science (WOS) and Scopus database. Every row represents an “OR” search operator. For the optimisation of the search queries, Wildcards and search operators were utilised. The search terms were optimised for both WOS and Scopus, below only the terms specific for the WOS database are shown. Two different Wildcards, “*” and “$”, were used, that represent unknown characters. The “*” Wildcard, retrieves any group of characters, including no character in a specific search term (*e.g.* Enterococc* represents both Enterococci and Enterococcus). “$” is equal to zero or one character, and is used to include all variations of a specific search term, (*e.g.* Orthop$edic would match results with Orhopedic and Orthopaedic). The Near/x operator is a proximity operator used to find words that appear within a specified number of characters of each other (*e.g.* Tissue Near/2 Communication would return articles with the terms Cell Communication and Communication between Cells).

| **Keywords** | **Bacteria** | **Bactericidal OR Antibacterial** | | | **Osteo OR Immune OR Osteoimmunology** | | | **Co-culture** | | | | | **Orthopedic biomaterial** | | |
| --- | --- | --- | --- | --- | --- | --- | --- | --- | --- | --- | --- | --- | --- | --- | --- |
| **Search terms** | Bacteri* | Bactericidal* | | | Osteo* | | | Co$culture | | | | | Orthop$edic | | |
|  | *Staphylococc** | *anti** | N/1 | Microbial* | Mesenchymal Stem Cell$ | | | Bacteri* | N/1 | Cell* | N/2 | Communication | Bone | | |
|  | *Aureus* |  |  | Bacteri* | Mesenchymal Stromal Cell$ | | |  |  | Tissue |  |  | Joint | | |
|  | *Epidermidis* |  |  | Biofilm | MSC$ | | |  |  | Material |  | Interaction | Biomaterial | | |
|  | *Enterococc** |  |  | Biofouling | Pre$osteoblast$ | | |  |  | Bone |  |  | Material | | |
|  | *Pseudomon** |  |  | Fouling | Osteoblast$ | | |  |  | Joint |  | Interface | Device | | |
|  | *Streptococc** |  |  | infect* | Osteoclast$ | | | Cell* |  | Implant |  |  | Implant | | |
|  | Methicillin-resistant *Staphilococcus Aureus* |  |  | sept* | Osteocyte$ | | |  |  | Device |  |  | Prosthetic | N/1 | Biomedical |
|  |  | Microbial* | N/1 | Resist* | Bone$cell$ | | |  |  | Biomaterial |  | Niche | Engineer* |  | Tissue |
|  | MRSA | Bacteri* |  |  | Immun* | | |  |  | Prosthetic |  |  |  |  | Bio* |
|  |  | Biofilm |  |  | Macrophag* | | |  |  | Orthop$edic |  |  |  |  |  |
|  |  | Sept* |  |  | Phagocyt* | | |  |  | Cellular |  | Cross$talk |  |  |  |
|  |  |  |  |  | Neutrophil$ | | |  |  | Inter* |  |  |  |  |  |
|  |  |  |  |  | Neutrocyte$ | | |  |  | Heterotypic* |  |  |  |  |  |
|  |  |  |  |  | Heterophil$ | | |  |  |  |  |  |  |  |  |
|  |  |  |  |  | Granulocyte$ | | |  |  |  |  |  |  |  |  |
|  |  |  |  |  | Monocyte$ | | |  |  |  |  |  |  |  |  |
|  |  |  |  |  | Dendritic Cell$ | | |  |  |  |  |  |  |  |  |
|  |  |  |  |  | Osteo$immun* | | |  |  |  |  |  |  |  |  |
|  |  |  |  |  | Foreign Body Response | | |  |  |  |  |  |  |  |  |
|  |  |  |  |  | FBR | | |  |  |  |  |  |  |  |  |
|  |  |  |  |  | Bone* | N/1 | Repair |  |  |  |  |  |  |  |  |
|  |  |  |  |  | Fracture |  | Healing |  |  |  |  |  |  |  |  |
|  |  |  |  |  |  |  | Regeneration |  |  |  |  |  |  |  |  |
|  |  |  |  |  | T$Cell$ | N/2 | Helper |  |  |  |  |  |  |  |  |
|  |  |  |  |  |  |  | Memory |  |  |  |  |  |  |  |  |
|  |  |  |  |  |  |  | Regulatory |  |  |  |  |  |  |  |  |
|  |  |  |  |  |  |  | Cytotoxic |  |  |  |  |  |  |  |  |

Table S2: An overview of the aim and results of all the included articles. **Abbreviations Ref:** References are ordered alphabetically. **Biomaterial:** Au: gold; BG: bioactive glass; CS: chitosan; ePTFE: expanded polytetrafluoroethylene; HA: hydroxyapatite; HACC: hydroxyapatite/calcium carbonate; Methylcellulose; PDMS: Polydimethylsiloxane; PLA: Polylactic acid; PLGA: poly(lactic-co-glycolic acid); PMMA: polymethyl methacrylate; SS: Stainless steel; Ti: Titanium; Ti6Al4V: Titanium alloy; TiO_2_: Titanium dioxide. **Surface modification:** Ag: Silver; AMP: antimicrobial peptide; APTES: (3-aminopropyl)triethoxysilane; APTMS: (3-aminopropyl)trimethoxysilane; Col1: collagen type 1; Cu: copper; DLCs: diamond-like carbon films; FN: fibronectin; GA: glutaraldehyde; IgG: immunoglobulin G; MgF_2_: Magnesium Fluoride; NPs: nanoparticles; PDA: polydopamine; PEG: polyethylene glycol; PHMB: poly (hexamethylene) biguanide hydrochloride; SLIPS: slippery liquid-infused porous surface; Si: Silicon; Sr: Strontium. **Cell seeding sequence:** b+c: simultaneous; b->c: bacteria first; c->b: cells first. **Model type:** osteo-bac: osteogenic cells-bacterial cells co-culture; im-bac: immune cells-bacterial cells co-culture. Gram-positive (+) or Gram-negative (-) bacteria are indicated in parentheses. **Others**: CFU: colony forming units; MOI: multiplicity of infection; MSCs: mesenchymal stem cells; PMNs: polymorphonuclear neutrophils; ROS: reactive oxygen species.

| **Ref** | **Biomaterial** | **Surface modification** | **Cell seeding sequence** | **Model type (Gram +/-)** | **Aim of the study** | **Results** |
| --- | --- | --- | --- | --- | --- | --- |
| (Boix-Lemonche et al., 2020) | Ti | AMP (BMAP27) | b->c | osteo-bac(+) | Investigation of functionalized titanium with antimicrobial peptide was conducted. | The surface coverage of osteoblasts was negatively influenced by bacteria, but this effect was significantly decreased by functionalized titanium. |
| (Boix-Lemonche et al., 2021) | Ti | AMP (BMAP27) | b->c | osteo-bac(+) | The bactericidal mechanism against *S. epidermidis* was characterized. | Surface coverage of osteoblasts was increased in comparison to mono-culture conditions. |
| (Chen et al., 2017) | ePTFE | SLIPS | b+c, b->c, c->b | im-bac(+) | An immobilized liquid surface was fabricated to minimize biofilm formation and adhesion on ePTFE. | Macrophage adhesion was reduced without compromising or enhancing their phagocytic activity. |
| (Chu et al., 2018) | PLGA, HA, HACC & combinations of these |  | b+c, b->c, c->b | osteo-bac(+) | Competitive colonization on a bifunctional biomaterial was investigated using different seeding sequences. | Successful surface colonization by osteoblasts was observed in co-culture, and the b->c model resulted in higher cell death compared to the c->b model. The choice of co-culture model had an impact on the outcome. |
| (Cicuéndez et al., 2018) | Methylcellulose | Levofloxacin | b+c | osteo-bac(+) | The effect of a scaffold on osteoblast and *S. aureus* survival was examined through a simple direct co-culture method to prevent and treat bacterial biofilm formation. | Levofloxacin release reduced osteoblast proliferation but improved survival during co-culture, and after 7 days of co-culture, there were no signs of infection. A high MOI (multiplicity of infection) was used due to levofloxacin's strong bactericidal properties. |
| (Cochis et al., 2020) | Ti6Al4V | BG, Sr ions, and Ag ions | b+c, b->c, c->b | osteo-bac(+) | The aim of this study was to develop antibacterial and bioactive surfaces. | Doped surfaces successfully inhibited bacteria and supported the survival of bone cells in both cells-first and bac-first models, but not in simultaneous models. |
| (Damiati et al., 2022) | TiO_2_ | Nanowires | c->b | osteo-bac(-) | The potential of high aspect ratio nano spikes or nanowires in promoting osteospecific differentiation of MSCs and preventing infection caused by biofilm-forming pathogens through bacterial killing was investigated. | A high aspect ratio protected MSCs from bacterial invasion, and the addition of a FN coating enhanced osteogenic regeneration. |
| (Deshmukh et al., 2016) | Glass | Ag NPs in HA | b+c, c->b | osteo-bac(-) | Competitive growth of bacterial and mammalian cells on silver-doped hydroxyapatite (Ag-HA) nanoparticle-coated glass slides was investigated. | Osteoblasts managed to outcompete bacteria for surface colonization in co-culture models with the coating. |
| (Ellett et al., 2019) | Glass | Microfluidic chamber | b->c (dynamic) | im-bac(+) | The effect of antibiotic pre-treatment on neutrophil recruitment and phagocytosis in a dynamic high throughput co-culture system was tested. | Neutrophil recruitment depended on the initial bacterial number and proliferation rates, with higher initial numbers resulting in greater recruitment. Neutrophils from patients with sepsis exhibited decreased recruitment. |
| (Foss et al., 2015) | Ti | CS | b+c | osteo-bac(+) | Antibacterial properties of a biomaterial with high roughness to enhance osteoblast integration and increase killing efficiency against bacteria were assessed using low and high MOI models. | SAOS-2 osteoblasts had higher surface coverage than *S. aureus* after 4 hours of co-culture. The presence of a high MOI had a significant effect on osteoblast attachment, with fewer osteoblasts attached to the surface after 4 hours compared to 30 minutes. |
| (Ghimire et al., 2016) | Ti | CS | c->b | osteo-bac(+) | A co-culture model was utilized to bridge the gap between *in vitro* and clinical post-operative infections. | Pre-attachment of osteoblasts had a reduced effect on bacterial colony-forming unit (CFU) count. At a low MOI, the material exhibited anti-infective properties after 30 minutes of co-culture. |
| (Gu et al., 2014) | CS |  | b->c (dynamic) | im-bac(+) | Real-time macrophage developments on chitosan and glass substrates during microfluidic culture were compared. | Macrophage cell mobility was higher on chitosan (41%) compared to glass (18%), and bacterial density on chitosan was significantly lower than on glass. |
| (Guo et al., 2017) | Ti | MgF_2_ NPs | b+c | im-bac(+) | The lysis and phagocytosis rate of isolated PMNs during co-culture on an antibacterial biomaterial were investigated in a culture medium and heparinized blood. | PMN stability increased when cultured in heparinized blood, and MgF_2_ NPs (nanoparticles) resulted in a higher phagocytosis rate and lower lysis. |
| (Hou et al., 2019) | Ti | FN, Col1, and Immunoglobulin G on DLCs | b->c | im-bac(+)(-) | The distinct influence of serum proteins adsorbed on biomaterial surfaces on initial bacterial adhesion, biofilm formation, and corresponding immune responses was investigated. | The recognition of Ig-G/FN proteins played a decisive role, with no noticeable physical effects observed. |
| (Jia et al., 2016) | Ti6Al4V | TiO_2_, PDA, and Ag NPs | c->b | osteo-bac(+) | The biomaterial aimed to restore defects regardless of the type of contamination by clearing pathogens first and orchestrating the healing process. | Osteogenic activity was improved, and no internal bacteria were found, indicating successful inhibition of bacterial growth. |
| (Jia et al., 2021) | Ti | Ag NPs | b+c | osteo-bac(+) | The antibacterial and osteogenic properties of core-shell silver nanoparticles were tested. | The scaffolds demonstrated 100 % anti-infection efficacy, as confirmed by counting detached bacteria. |
| (Lee et al., 2011) | PDMS | 100 μg/mL FN | b->c (dynamic) | osteo-bac(+) | The effect of different metabolically active bacteria on the behaviour and growth of osteoblasts within a microfluidic channel was studied. | A 3D structure with nodules inside the entire channel was observed after 12 days in the control study. Resistant bacteria prevented preostosteoblasts (MC3T3-E1) from forming 3D layers, while the osteoblasts stood a chance against other bacteria. |
| (Li et al., 2017) | Glass | APTES/APTM, GA, Col1 and PDMS | c->b | im-bac(-) | The differences in antibacterial and phagocytic properties between collagen-coated glass and PDMS substrates were presented. | Lower bacterial CFU count was observed inside macrophages on APTES/APTMS + GA + Col1 surfaces, indicating a decreased phagocytic response and invasion rate. |
| (Martínez-Pérez et al., 2019) | Ti6Al4V | Nanoporous surface | b+c | osteo-bac(+) | Nanostructured surfaces were compared for their adherence, osseointegration, and antibacterial properties. | The presence of preosteoblasts increased adherence to all bacterial strains. |
| (Mendoza et al., 2017) | Au NPs | CS | c->b | im-bac(+)(-) | To obtain an ideal *in vitro* model where macrophages are not negatively influenced by the material. | The addition of NPs (nanoparticles) reduced the number of living intracellular bacteria. |
| (Mohiti-Asli et al., 2016) | PLA nanofibers | AgNO3 NPs | c->b | osteo-bac(+) | The efficacy of scaffolds in treating or preventing osteomyelitis was determined. | The scaffolds inhibited early biofilm formation during co-culture, but bacterial growth increased rapidly after 6 days. The scaffold also supported osteogenesis. |
| (Qiu et al., 2019) | Ti | Graphene oxide and minocycline | c->b | osteo-bac(+) | The antibacterial activity and osteogenic properties were tested in the presence of *S. aureus* and rat bone mesenchymal stem cells in *in vitro* and *in vivo* environments. | The material exhibited high antibacterial properties, and the anti-inflammatory effect of minocycline created a suitable microenvironment for osteogenic differentiation and tissue regeneration. |
| (Reigada et al., 2020) | Ti | Low density polyethylene | b+c, c->b | im-bac(+) | Biofilm formation on orthopaedic implants or endotracheal tubes was simulated through *S. aureus* and neutrophil (HL-60) co-culture. | Based on CFU counting and SEM imaging, the biomaterial significantly inhibited bacterial growth, and the presence of leukocytes further decreased bacterial viability. |
| (Rivera et al., 2021) | SS | Zein, BG and Cu | c->b | osteo-bac(+)(-) | The efficiency of an antibacterial and pro-angiogenic material was tested. | The zein/Cu-BG combination had a significant antibacterial effect and enhanced cell viability compared to the control samples. |
| (Sánchez-Salcedo et al., 2023) | BG | Ag NPs | c->b | osteo-bac(+) | The antibacterial and cytotoxic potential of Ag/mesoporous BG nanocomposites was evaluated. | During co-culture, osteoblasts maintained normal morphology, their numbers were minimally affected, and the bacterial count decreased significantly. |
| (Shen et al., 2020) | Ti | Si/Cu doped porous coating | b+c | osteo-bac(+) | Assessment of functionalized titanium with antibacterial and osteogenic properties was conducted. | *S. mutans* could grow on top of osteoblasts on control surfaces, but Si/Cu-doped substrates ensured the survival of preosteoblasts (MC3T3-E1) in the presence of *S. mutans* and promoted osteogenic differentiation. |
| (Svensson et al., 2014) | Au | Au NPs and Au sputtered Si wafer | c->b | im-bac(+) | Adhesion and biofilm formation of *S. epidermidis* and human monocyte activation on smooth and nanostructured gold surfaces were studied individually and in co-culture. | Smooth gold (Au) supported more living host cells compared to nanostructured gold, with no difference in dead bacteria. Both forms of gold were involved in specific inflammatory events when exposed to phagocytic prey. |
| (Tan et al., 2020) | Ti | MgO | b->c | osteo-bac(+)(-) | The selective antibacterial activity of MgO samples was evaluated. | MgO films exhibited no cytotoxicity to osteoblast cells, selectively killed bacteria, and promoted cell proliferation in co-culture. |
| (Tao et al., 2019) | Ti | Nanotube BMP-2 & gentamycin carriers | b+c | osteo-bac(+)(-) | The antibacterial ability and osteogenic properties of Ti substrate were improved. | Almost no *S. aureus* was observed on top of osteoblasts during co-culture using SEM imaging. |
| (Tran and Tran, 2021) | Ti | AgNPs and α-amylase | b+c, c->b | osteo-bac(+) | *In vitro* study was conducted to prevent and treat biofilm formation on a biomaterial using b+c and c->b methods. | Bacteria survived on top of osteoblasts in co-culture and were resistant to antibiotic treatment. In the c->b co-culture, *S. aureus* preferred attaching to osteoblasts, improving its own survival through internalization. |
| (Wagner and Bryers, 2004) | PEG-g-PDA polymer | Cell adhesion peptides, monoclonal antibodies or macrophage integrin receptors | c->b | im-bac(+)(-) | The interaction between macrophages and bacteria on an immunomodulation biomaterial was investigated. | Pro-inflammatory response was higher on all surfaces in the presence of bacteria or lipopolysaccharide. Macrophages adhering to antibody fragment-modified surfaces exhibited sustained enhanced phagocytic response and higher bacterial killing efficiency. |
| (Yang et al., 2021) | Ti | UV/OZONE treatment | b+c, b->c, c->b (in-direct) | im-bac(+) | The immune and indirect osteoimmune effects of infected UV/Ozone treated surfaces were examined. | The c->b model showed increased phagocytosis of *S. aureus* by macrophages. *S. aureus* was also observed on top of macrophages. In the c+b and b->c models, more *S. aureus* adhered to the sample surfaces, and fewer bacteria were phagocytosed. The conditioned medium of im-bac co-culture with an enhanced pro-inflammatory response improved the osteogenic response of MSCs. |
| (Yue et al., 2014) | Ti & Ti alloys | photocatalytic activated anodized nano surfaces | b->c (dynamic) | osteo-bac(+) | The effect of human-bone-marrow-mesenchymal-stem (MSCs) cells and osteosarcoma cells (U2Os) on photocatalytic activation of anodized TiO_2_ surfaces in parallel flow chambers was investigated. | MSCs did not survive on untreated surfaces in the presence of *S. epidermidis* or *S. aureus*. However, when bacteria were killed by treated samples within the first 60 minutes, MSCs survived. |
| (Zaatreh et al., 2016) | Ti6Al4V, polystyrene and PMMA bone cement |  | c->b | osteo-bac(+) | An *in vitro* co-culture setup of human primary osteoblasts and *S. epidermidis* was established to model the onset of implant-associated infections and analyse antimicrobial implant surfaces and coatings. | The presence of osteoblasts hindered the working mechanisms of antibacterial surfaces. *S. epidermidis* formed clusters on top of the cells, and more planktonic bacteria were present. The antibiotic effect was stronger on osteoblasts than on reducing the *S. epidermidis* population. |
| (Zaatreh et al., 2017) | Ti6Al4V | Mg | c->b | osteo-bac(+) | The antibacterial and cytotoxic surface properties of these surfaces were investigated. | *S. epidermidis* on top of osteoblasts were protected against the antibacterial surface, and the antibacterial effect was reduced in co-culture. |
| (Zwicker et al., 2022) | Ti6Al4V | PHMB (<10nm) | b->c | im-bac(+)(-) | Prevention of the foreign body response (FBR) was studied to limit the pro-inflammatory response of macrophages against a bactericidal implant. | iROS generation and cytokine secretion were highest after 24 hours, with no significant differences between mono and co-culture models. The coating enabled macrophage attachment and viability through early bacterial killing. |

Table S3: An overview of the experimental analysis methods used to evaluate osteo-bac co-cultures. Gram-positive (+) or Gram-negative (-) bacteria are indicated in parentheses.

|  | **Osteo-bac co-cultures** | **(+)** | | | | | | | | | | | | | | | | | **(-)** | **(+) & (-)** | | | **(+) dynamic** | |
| --- | --- | --- | --- | --- | --- | --- | --- | --- | --- | --- | --- | --- | --- | --- | --- | --- | --- | --- | --- | --- | --- | --- | --- | --- |
| **Viability and proliferation** | CFU count |  |  |  |  | X | X | X |  | X |  | X |  |  |  | X | X | X |  | X |  |  | X |  |
|  | Cell count |  |  |  |  | X | X | X |  |  | X |  |  |  |  |  | X | X |  | X |  |  |  |  |
|  | Colorimetric |  |  |  |  |  |  |  |  |  |  |  |  |  |  |  |  |  | X |  |  |  |  |  |
|  | Gentamycin protocol |  |  |  |  |  |  |  | X |  |  |  |  |  |  | X |  |  |  |  |  |  |  |  |
|  | ROS |  |  |  |  |  |  |  |  |  |  |  |  |  |  |  |  |  |  |  |  |  |  |  |
|  | LDH |  |  | X |  |  |  |  |  |  |  |  |  |  |  |  |  |  |  |  |  |  |  |  |
| **Microscopy and staining** | SEM |  |  | X |  |  | X |  | X |  |  | X | X |  | X | X | X | X | X | X | X |  |  |  |
|  | Light microscopy |  |  |  |  |  |  |  | X |  |  |  |  |  |  | X |  |  |  |  |  |  | X |  |
|  | Cytoskeletal staining | X | X | X | X |  | X |  | X | X |  |  | X |  |  |  |  |  |  |  |  | X |  | X |
|  | Nucleus staining (Hoechst/DAPI) | X | X | X | X |  |  |  | X | X |  |  |  | X |  |  |  |  |  |  |  | X |  | X |
|  | Bacterial staining |  |  |  |  |  | X |  |  |  |  |  |  |  |  |  |  |  |  |  |  |  |  |  |
|  | Acridine Orange |  |  | X |  |  |  |  |  |  | X |  |  | X |  |  |  |  |  |  |  |  |  |  |
|  | Live/dead staining |  |  | X |  |  |  |  |  |  |  |  |  |  | X | X |  |  | X |  | X |  | X |  |
|  | Other |  |  |  |  |  |  |  |  |  |  |  |  |  |  |  |  |  | X |  |  |  |  |  |
| **Flow cytometry and gene or protein analysis** | Flow cytometry |  |  |  |  |  |  |  | X |  |  |  |  |  |  |  |  |  |  |  |  |  |  |  |
|  | qPCR |  |  |  |  |  |  |  |  |  |  |  |  |  |  |  |  |  | X |  |  |  |  |  |
|  | ELISA |  |  |  |  |  |  |  |  |  |  |  |  |  |  |  |  |  |  |  |  |  |  |  |
| **Osteogenic differentiation** | Runx2 |  |  |  |  |  |  |  |  |  |  |  |  |  | X |  |  |  |  |  |  |  |  |  |
|  | ALP |  |  |  |  |  |  |  |  |  |  |  |  |  | X |  |  |  |  |  |  |  |  |  |
|  | OCN |  |  |  |  |  |  |  |  |  |  | X |  |  | X |  |  |  |  |  |  |  | X |  |
|  | Mineralization (Calcium or ARS) |  |  | X | X |  |  |  |  |  |  | X |  |  |  |  |  |  |  |  |  |  | X |  |
|  | Reference | (Boix-Lemonche et al., 2020) | (Boix-Lemonche et al., 2021) | (Chu et al., 2018) | (Cicuéndez et al., 2018) | (Cochis et al., 2020) | (Foss et al., 2015) | (Ghimire et al., 2016) | (Jia et al., 2016) | (Jia et al., 2021) | (Martínez-Pérez et al., 2019) | (Mohiti-Asli et al., 2016) | (Qiu et al., 2019) | (Sánchez-Salcedo et al., 2023) | (Shen et al., 2020) | (Tran and Tran, 2021) | (Zaatreh et al., 2016) | (Zaatreh et al., 2017) | (Damiati et al., 2022) | (Rivera et al., 2021) | (Tan et al., 2020) | (Tao et al., 2019) | (Lee et al., 2011) | (Yue et al., 2014) |

Table S4: An overview of the experimental analysis methods used to evaluate im-bac co-cultures. Gram-positive (+) or Gram-negative (-) bacteria are indicated in parentheses.

|  | **Im-bac co-cultures** | **(+)** | | | | | **(-)** | | **(+) & (-)** | | | | **(+) dynamic** | |  |  |  |  |  |  |  |  |  |  |
| --- | --- | --- | --- | --- | --- | --- | --- | --- | --- | --- | --- | --- | --- | --- | --- | --- | --- | --- | --- | --- | --- | --- | --- | --- |
| **Viability and proliferation** | CFU count | X | X | X |  |  | X | X |  | X | X | X |  | X |  |  |  |  |  |  |  |  |  |  |
|  | Cell count |  |  |  | X |  |  |  |  |  |  |  |  | X |  |  |  |  |  |  |  |  |  |  |
|  | Colorimetric |  |  |  |  |  |  |  |  |  |  | X |  |  |  |  |  |  |  |  |  |  |  |  |
|  | Gentamycin protocol |  |  |  |  |  |  | X |  | X |  |  |  |  |  |  |  |  |  |  |  |  |  |  |
|  | ROS |  |  |  | X | X |  |  |  |  | X |  | X |  |  |  |  |  |  |  |  |  |  |  |
|  | LDH |  | X |  | X |  |  |  |  |  |  |  |  |  |  |  |  |  |  |  |  |  |  |  |
| **Microscopy and staining** | SEM |  |  | X | X | X |  |  | X | X |  |  |  |  |  |  |  |  |  |  |  |  |  |  |
|  | Light-microscopy |  |  |  |  | X | X |  |  |  |  |  |  | X |  |  |  |  |  |  |  |  |  |  |
|  | cytoskeletal staining |  |  |  |  |  |  |  |  |  |  | X |  |  |  |  |  |  |  |  |  |  |  |  |
|  | nucleus staining (Hoechst/DAPI) |  |  |  |  |  |  |  |  |  | X |  |  |  |  |  |  |  |  |  |  |  |  |  |
|  | Bacterial staining | X |  |  |  |  |  |  |  |  | X |  |  |  |  |  |  |  |  |  |  |  |  |  |
|  | Acridine Orange |  |  |  |  |  |  |  |  |  |  |  |  |  |  |  |  |  |  |  |  |  |  |  |
|  | Live/dead staining |  |  |  |  | X |  |  |  | X | X |  |  |  |  |  |  |  |  |  |  |  |  |  |
|  | Other | X | X |  |  | X |  |  |  |  |  |  | X |  |  |  |  |  |  |  |  |  |  |  |
| **Flow cytometry and gene or protein analysis** | Flow cytometry | X |  |  |  |  |  |  |  | X |  |  |  |  |  |  |  |  |  |  |  |  |  |  |
|  | qPCR |  |  |  | X |  |  |  |  |  | X | X |  |  |  |  |  |  |  |  |  |  |  |  |
|  | ELISA |  |  |  | X |  |  |  |  |  | X | X |  |  |  |  |  |  |  |  |  |  |  |  |
| **Osteogenic differentiation** | Runx2 |  |  |  |  |  |  |  |  |  |  |  |  |  |  |  |  |  |  |  |  |  |  |  |
|  | ALP |  |  |  |  |  |  |  |  |  |  |  |  |  |  |  |  |  |  |  |  |  |  |  |
|  | OCN |  |  |  |  |  |  |  |  |  |  |  |  |  |  |  |  |  |  |  |  |  |  |  |
|  | Mineralization (Calcium or ARS) |  | X |  | X |  |  |  |  |  |  |  |  |  |  |  |  |  |  |  |  |  |  |  |
|  | Reference | (Chen et al., 2017) | (Guo et al., 2017) | (Reigada et al., 2020) | (Svensson et al., 2014) | (Yang et al., 2021) | (Deshmukh et al., 2016) | (Li et al., 2017) | (Hou et al., 2019) | (Mendoza et al., 2017) | (Wagner and Bryers, 2004) | (Zwicker et al., 2022) | (Ellett et al., 2019) | (Gu et al., 2014) |  |  |  |  |  |  |  |  |  |  |

**References**

Boix-Lemonche, G., Guillem-Marti, J., D’Este, F., Manero, J. M., and Skerlavaj, B. (2020). Covalent grafting of titanium with a cathelicidin peptide produces an osteoblast compatible surface with antistaphylococcal activity. *Colloids Surfaces B Biointerfaces* 185, 110586. doi: 10.1016/J.COLSURFB.2019.110586.

Boix-Lemonche, G., Guillem-Marti, J., Lekka, M., D’Este, F., Guida, F., Manero, J. M., et al. (2021). Membrane perturbation, altered morphology and killing of Staphylococcus epidermidis upon contact with a cytocompatible peptide-based antibacterial surface. *Colloids Surfaces B Biointerfaces* 203, 111745. doi: 10.1016/j.colsurfb.2021.111745.

Chen, J., Howell, C., Haller, C. A., Patel, M. S., Ayala, P., Moravec, K. A., et al. (2017). An immobilized liquid interface prevents device associated bacterial infection in vivo. *Biomaterials* 113, 80–92. doi: 10.1016/j.biomaterials.2016.09.028.

Chu, L., Yang, Y., Yang, S., Fan, Q., Yu, Z., Hu, X.-L., et al. (2018). Preferential Colonization of Osteoblasts Over Co-cultured Bacteria on a Bifunctional Biomaterial Surface. *Front. Microbiol.* 9, 1–13. doi: 10.3389/fmicb.2018.02219.

Cicuéndez, M., Doadrio, J. C., Hernández, A., Portolés, M. T., Izquierdo-Barba, I., and Vallet-Regí, M. (2018). Multifunctional pH sensitive 3D scaffolds for treatment and prevention of bone infection. *Acta Biomater.* 65, 450–461. doi: 10.1016/j.actbio.2017.11.009.

Cochis, A., Barberi, J., Ferraris, S., Miola, M., Rimondini, L., Vernè, E., et al. (2020). Competitive Surface Colonization of Antibacterial and Bioactive Materials Doped with Strontium and/or Silver Ions. *Nanomaterials* 10, 120. doi: 10.3390/nano10010120.

Damiati, L. A., Tsimbouri, M. P., Hernandez, V.-L., Jayawarna, V., Ginty, M., Childs, P., et al. (2022). Materials-driven fibronectin assembly on nanoscale topography enhances mesenchymal stem cell adhesion, protecting cells from bacterial virulence factors and preventing biofilm formation. *Biomaterials* 280, 121263. doi: 10.1016/j.biomaterials.2021.121263.

Deshmukh, K. R., Ramanan, S. R., and Kowshik, M. (2016). Low-temperature-processed biocompatible Ag-HAp nanoparticles with anti-biofilm efficacy for tissue engineering applications. *J. Sol-Gel Sci. Technol.* 80, 738–747. doi: 10.1007/S10971-016-4149-2/FIGURES/8.

Ellett, F., Jalali, F., Marand, A. L., Jorgensen, J., Mutlu, B. R., Lee, J., et al. (2019). Microfluidic arenas for war games between neutrophils and microbes. *Lab Chip* 19, 1205–1216. doi: 10.1039/C8LC01263F.

Foss, B. L., Ghimire, N., Tang, R., Sun, Y., and Deng, Y. (2015). Bacteria and osteoblast adhesion to chitosan immobilized titanium surface: A race for the surface. *Colloids Surfaces B Biointerfaces* 134, 370–376. doi: 10.1016/j.colsurfb.2015.07.014.

Ghimire, N., Foss, B. L., Sun, Y., and Deng, Y. (2016). Interactions among osteoblastic cells, Staphylococcus aureus , and chitosan-immobilized titanium implants in a postoperative coculture system: An in vitro study. *J. Biomed. Mater. Res. Part A* 104, 586–594. doi: 10.1002/jbm.a.35597.

Gu, Y., Zhang, W., Wang, H., and Lee, W. Y. (2014). Chitosan surface enhances the mobility, cytoplasm spreading, and phagocytosis of macrophages. *Colloids Surfaces B Biointerfaces* 117, 42–50. doi: 10.1016/j.colsurfb.2014.01.051.

Guo, G., Zhou, H., Wang, Q., Wang, J., Tan, J., Li, J., et al. (2017). Nano-layered magnesium fluoride reservoirs on biomaterial surfaces strengthen polymorphonuclear leukocyte resistance to bacterial pathogens. *Nanoscale* 9, 875–892. doi: 10.1039/C6NR07729C.

Hou, W., Liu, Y., Wu, S., Zhang, H., Guo, B., Zhang, B., et al. (2019). Preadsorption of Serum Proteins Regulates Bacterial Infections and Subsequent Macrophage Phagocytosis on Biomaterial Surfaces. *ACS Appl. Bio Mater.* 2, 5957–5964. doi: 10.1021/ACSABM.9B00890.

Jia, Z., Wen, M., Xiong, P., Yan, J., Zhou, W., Cheng, Y., et al. (2021). Mussel bioinspired morphosynthesis of substrate anchored core–shell silver self-assemblies with multifunctionality for bioapplications. *Mater. Sci. Eng. C* 123, 112025. doi: 10.1016/j.msec.2021.112025.

Jia, Z., Xiu, P., Xiong, P., Zhou, W., Cheng, Y., Wei, S., et al. (2016). Additively Manufactured Macroporous Titanium with Silver-Releasing Micro-/Nanoporous Surface for Multipurpose Infection Control and Bone Repair - A Proof of Concept. *ACS Appl. Mater. Interfaces* 8, 28495–28510. doi: 10.1021/ACSAMI.6B10473/SUPPL_FILE/AM6B10473_SI_001.PDF.

Lee, J.-H., Wang, H., Kaplan, J. B., and Lee, W. Y. (2011). Microfluidic Approach to Create Three-Dimensional Tissue Models for Biofilm-Related Infection of Orthopaedic Implants. *Tissue Eng. Part C Methods* 17, 39–48. doi: 10.1089/ten.tec.2010.0285.

Li, C., Ding, Y., Kuddannaya, S., Zhang, Y., and Yang, L. (2017). Anti-bacterial properties of collagen-coated glass and polydimethylsiloxane substrates. *J. Mater. Sci.* 52, 9963–9978. doi: 10.1007/s10853-017-1175-6.

Martínez-Pérez, M., Conde, A., Arenas, M.-A., Mahíllo-Fernandez, I., De-Damborenea, J.-J., Pérez-Tanoira, R., et al. (2019). The “Race for the Surface” experimentally studied: In vitro assessment of Staphylococcus spp. adhesion and preosteoblastic cells integration to doped Ti-6Al-4V alloys. *Colloids Surfaces B Biointerfaces* 173, 876–883. doi: 10.1016/j.colsurfb.2018.10.076.

Mendoza, G., Regiel-Futyra, A., Andreu, V., Sebastián, V., Kyzioł, A., Stochel, G., et al. (2017). Bactericidal Effect of Gold-Chitosan Nanocomposites in Coculture Models of Pathogenic Bacteria and Human Macrophages. *ACS Appl. Mater. Interfaces* 9, 17693–17701. doi: 10.1021/acsami.6b15123.

Mohiti-Asli, M., Molina, C., Diteepeng, T., Pourdeyhimi, B., and Loboa, E. G. (2016). Evaluation of Silver Ion-Releasing Scaffolds in a 3D Coculture System of MRSA and Human Adipose-Derived Stem Cells for Their Potential Use in Treatment or Prevention of Osteomyelitis. *Tissue Eng. Part A* 22, 1258–1263. doi: 10.1089/ten.tea.2016.0063.

Qiu, J., Qian, W., Zhang, J., Chen, D., Yeung, K. W. K., and Liu, X. (2019). Minocycline hydrochloride loaded graphene oxide enables enhanced osteogenic activity in the presence of Gram-positive bacteria, Staphylococcus aureus. *J. Mater. Chem. B* 7, 3590–3598. doi: 10.1039/C9TB00405J.

Reigada, I., Guarch-Pérez, C., Patel, J. Z., Riool, M., Savijoki, K., Yli-Kauhaluoma, J., et al. (2020). Combined Effect of Naturally-Derived Biofilm Inhibitors and Differentiated HL-60 Cells in the Prevention of Staphylococcus aureus Biofilm Formation. *Microorganisms* 8, 1757. doi: 10.3390/microorganisms8111757.

Ribeiro, M., Monteiro, F. J., and Ferraz, M. P. (2012). Infection of orthopedic implants with emphasis on bacterial adhesion process and techniques used in studying bacterial-material interactions. *Biomatter* 2, 176–194. doi: 10.4161/biom.22905.

Rivera, L. R., Cochis, A., Biser, S., Canciani, E., Ferraris, S., Rimondini, L., et al. (2021). Antibacterial, pro-angiogenic and pro-osteointegrative zein-bioactive glass/copper based coatings for implantable stainless steel aimed at bone healing. *Bioact. Mater.* 6, 1479–1490. doi: 10.1016/j.bioactmat.2020.11.001.

Sánchez-Salcedo, S., García, A., González-Jiménez, A., and Vallet-Regí, M. (2023). Antibacterial effect of 3D printed mesoporous bioactive glass scaffolds doped with metallic silver nanoparticles. *Acta Biomater.* 155, 654–666. doi: 10.1016/j.actbio.2022.10.045.

Shen, X., Hu, W., Ping, L., Liu, C., Yao, L., Deng, Z., et al. (2020). Antibacterial and Osteogenic Functionalization of Titanium With Silicon/Copper-Doped High-Energy Shot Peening-Assisted Micro-Arc Oxidation Technique. *Front. Bioeng. Biotechnol.* 8, 1138. doi: 10.3389/FBIOE.2020.573464/BIBTEX.

Svensson, S., Forsberg, M., Hulander, M., Vazirisani, F., Thomsen, P., Trobos, M., et al. (2014). Role of nanostructured gold surfaces on monocyte activation and Staphylococcus epidermidis biofilm formation. *Int. J. Nanomedicine* 9, 775. doi: 10.2147/IJN.S51465.

Tan, J., Liu, Z., Wang, D., Zhang, X., Qian, S., and Liu, X. (2020). A facile and universal strategy to endow implant materials with antibacterial ability via alkalinity disturbing bacterial respiration. *Biomater. Sci.* 8, 1815–1829. doi: 10.1039/C9BM01793C.

Tao, B., Deng, Y., Song, L., Ma, W., Qian, Y., Lin, C., et al. (2019). BMP2-loaded titania nanotubes coating with pH-responsive multilayers for bacterial infections inhibition and osteogenic activity improvement. *Colloids Surfaces B Biointerfaces* 177, 242–252. doi: 10.1016/j.colsurfb.2019.02.014.

Tran, H. A., and Tran, P. A. (2021). In Situ Coatings of Silver Nanoparticles for Biofilm Treatment in Implant-Retention Surgeries: Antimicrobial Activities in Monoculture and Coculture. *ACS Appl. Mater. Interfaces* 13, 41435–41444. doi: 10.1021/acsami.1c08239.

Wagner, V. E., and Bryers, J. D. (2004). Poly(ethylene glycol)-polyacrylate copolymers modified to control adherent monocyte-macrophage physiology: Interactions with attachingStaphylococcus epidermidis orPseudomonas aeruginosa bacteria. *J. Biomed. Mater. Res.* 69A, 79–90. doi: 10.1002/jbm.a.20115.

Yang, Y., Zhang, H., Komasa, S., Morimoto, Y., Sekino, T., Kawazoe, T., et al. (2021). UV/ozone irradiation manipulates immune response for antibacterial activity and bone regeneration on titanium. *Mater. Sci. Eng. C* 129, 112377. doi: 10.1016/j.msec.2021.112377.

Yue, C., Kuijer, R., Kaper, H. J., van der Mei, H. C., and Busscher, H. J. (2014). Simultaneous interaction of bacteria and tissue cells with photocatalytically activated, anodized titanium surfaces. *Biomaterials* 35, 2580–2587. doi: 10.1016/j.biomaterials.2013.12.036.

Zaatreh, S., Haffner, D., Strauss, M., Dauben, T., Zamponi, C., Mittelmeier, W., et al. (2017). Thin magnesium layer confirmed as an antibacterial and biocompatible implant coating in a co‑culture model. *Mol. Med. Rep.* 15, 1624–1630. doi: 10.3892/mmr.2017.6218.

Zaatreh, S., Wegner, K., Strauß, M., Pasold, J., Mittelmeier, W., Podbielski, A., et al. (2016). Co-Culture of S. epidermidis and Human Osteoblasts on Implant Surfaces: An Advanced In Vitro Model for Implant-Associated Infections. *PLoS One* 11, e0151534. doi: 10.1371/journal.pone.0151534.

Zwicker, P., Schmidt, T., Hornschuh, M., Lode, H., Kramer, A., and Müller, G. (2022). In vitro response of THP-1 derived macrophages to antimicrobially effective PHMB-coated Ti6Al4V alloy implant material with and without contamination with S. epidermidis and P. aeruginosa. *Biomater. Res.* 26, 1. doi: 10.1186/s40824-021-00247-1.
